# Deep learning-based diagnosis of Alzheimer’s disease using brain magnetic resonance images: an empirical study

**DOI:** 10.1038/s41598-022-22917-3

**Published:** 2022-10-26

**Authors:** Jun Sung Kim, Ji Won Han, Jong Bin Bae, Dong Gyu Moon, Jin Shin, Juhee Eliana Kong, Hyungji Lee, Hee Won Yang, Eunji Lim, Jun Yup Kim, Leonard Sunwoo, Se Jin Cho, Dongsoo Lee, Injoong Kim, Sang Won Ha, Min Ju Kang, Chong Hyun Suh, Woo Hyun Shim, Sang Joon Kim, Ki Woong Kim

**Affiliations:** 1 Institute of Human Behavioral Medicine, Seoul National University Medical Research Center, Seoul, Republic of Korea; 2 Department of Neuropsychiatry, Seoul National University Bundang Hospital, 82, Gumi-Ro 173 Beon-Gil, Bundang-Gu, Seongnam-Si, Gyeonggi-Do 13620 Republic of Korea; 3 Department of Psychiatry, Seoul National University College of Medicine, Seoul, Republic of Korea; 4 Department of Psychiatry, Chungnam National University Hospital, Daejeon, Republic of Korea; 5 Department of Neuropsychiatry, Gyeongsang National University Changwon Hospital, Changwon, Republic of Korea; 6 Department of Neurology, Seoul National University Bundang Hospital, Seongnam, Republic of Korea; 7 Department of Radiology, Seoul National University Bundang Hospital, Seongnam, Republic of Korea; 8 Department of Radiology, Seoul National University College of Medicine, Seoul, Republic of Korea; 9 VUNO Inc., Seoul, Republic of Korea; 10 Department of Radiology, Veterans Health Service Medical Center, Seoul, Republic of Korea; 11 Department of Neurology, Veterans Health Service Medical Center, Seoul, Republic of Korea; 12 Department of Radiology and Research Institute of Radiology, Asan Medical Center, University of Ulsan College of Medicine, Seoul, Republic of Korea; 13 Department of Brain and Cognitive Science, Seoul National University College of Natural Science, Seoul, Republic of Korea

**Keywords:** Diagnosis, Medical imaging

## Abstract

The limited accessibility of medical specialists for Alzheimer’s disease (AD) can make obtaining an accurate diagnosis in a timely manner challenging and may influence prognosis. We investigated whether VUNO Med-DeepBrain AD (DBAD) using a deep learning algorithm can be employed as a decision support service for the diagnosis of AD. This study included 98 elderly participants aged 60 years or older who visited the Seoul Asan Medical Center and the Korea Veterans Health Service. We administered a standard diagnostic assessment for diagnosing AD. DBAD and three panels of medical experts (ME) diagnosed participants with normal cognition (NC) or AD using T1-weighted magnetic resonance imaging. The accuracy (87.1% for DBAD and 84.3% for ME), sensitivity (93.3% for DBAD and 80.0% for ME), and specificity (85.5% for DBAD and 85.5% for ME) of both DBAD and ME for diagnosing AD were comparable; however, DBAD showed a higher trend in every analysis than ME diagnosis. DBAD may support the clinical decisions of physicians who are not specialized in AD; this may enhance the accessibility of AD diagnosis and treatment.

## Introduction

The number of people with Alzheimer’s disease (AD) is estimated to be more than 50 million worldwide^1^ and is expected to increase more than three-fold within the next 30 years^2^. However, in many regions of the world, it is a challenge to obtain an accurate diagnosis in a timely manner owing to limited accessibility to AD medical specialists. For example, hospitals that offer specialized diagnosis and management of AD are usually concentrated in urban areas, although AD is more prevalent in rural areas^3^. This rural–urban inequality in accessibility to specialists may contribute to disparities in health outcomes of people with AD between rural and urban areas^4^.

Structural neuroimaging, such as brain magnetic resonance imaging (MRI), is an essential test for diagnosing AD and monitoring its course^5^. According to the Organization for Economic Co-operation and Development, the number of MRI units is constantly increasing worldwide^6^. However, in many regions, the results of brain MRI cannot be interpreted in a timely and accurate manner owing to a lack of AD specialists. Moreover, even AD specialists cannot identify the early changes in AD that occur on brain MRI with the naked eye^7,8^. A technology that can accurately identify people with AD from the prodromal or early dementia stages using brain MRI would increase the diagnostic rate of AD and advance the time of diagnosis of AD, thus reducing the health outcome disparities between regions by supporting the clinical decision of physicians who are not specialized in AD.

Deep learning (DL) using convolutional neural networks (CNN) has been proposed as a promising tool for supporting clinical decisions on digital brain images of AD^9–15^. In many previous studies, three-dimensional (3D) brain MR images were employed as input data in DL algorithms for diagnosing AD^10,12–14,16–18^. However, 3D brain MRI is not available in most clinical settings because 3D MRI sequences have longer acquisition times than two-dimensional (2D) MRI sequences^19^, which significantly increase computational burden, storage, and cost^20,21^. Thus, the previously developed DL algorithms for diagnosing AD using 3D MRI may be difficult to apply to most brain MRI scans obtained in typical clinical settings. Therefore, we developed VUNO Med-DeepBrain AD, version 1.0.0 (DBAD; VUNO Inc., Seoul, Korea), which is the first convolutional neural network-based model for diagnosing AD using 2D brain MR images as input data, and demonstrated its diagnostic accuracy for AD to be excellent in both Caucasians and Asians^22^.

This study aimed to investigate whether VUNO Med-DeepBrain AD using 2D brain MRI can be employed as a decision support service for AD diagnoses in hospitals, by comparing the diagnostic performance of VUNO Med-DeepBrain AD with that of AD medical specialists working at referral hospitals. If VUNO Med-DeepBrain AD would be found to be as accurate as the decision of medical specialists, it can be easily implemented in real clinical settings because it has fast processing speed and uses 2D images that are commonly employed in usual clinical settings. Introduction of VUNO Med-DeepBrain AD may contribute to the early diagnosis of AD not only in memory clinics but also in any medical settings that uses brain MRI.

## Methods

### Participants

We enrolled 100 older adults aged 60 or older (34 with a history of AD and 66 without a history of AD) from the visitors to the Seoul Asan Medical Center (AMC) and the Korea Veterans Health Service (KVHS). Then we administered a standard diagnostic assessment for AD to 98 participants of them at the Seoul National University Bundang Hospital (SNUBH) after excluding two participants who refused the standard diagnostic assessment. All participants and/or their legal guardians provided a written informed consent to participate in this study. We obtained ethics approval from the Institutional Review Board of AMC, KVHS and SNUBH. All experiments were performed in accordance with relevant guidelines and regulations.

### Acquisition and preprocessing of brain MRI

We acquired 3D T1-weighted MR images in the Digital Imaging and Communications in Medicine format using a 3.0 Tesla Ingenia scanner (Philips Medical Systems; Eindhoven, NL) at the AMC and a 3.0 Tesla Magnetom Skyra (Siemens Healthineers, Erlangen, Germany) or a 3.0 Tesla Magnetom Vida scanner (Siemens Healthineers) at the KVHS. The parameters were as follows: repetition time = 9.6 ms, echo time = 4.6 ms, flip angle = 8°, field of view = 224 × 224 mm^2^, slice thickness = 1 mm with no gap, and matrix size = 224 × 224 mm^2^ in the Ingenia scanner; repetition time = 1900 ms, echo time = 2.6 ms, flip angle = 9°, field of view = 230 × 230 mm^2^, slice thickness = 1 mm, and matrix size = 256 × 256 mm^2^ in the Magnetom Skyra scanner; and repetition time = 1900 ms, echo time = 2.9 ms, flip angle = 9°, field of view = 230 × 230 mm^2^, slice thickness = 1 mm, and matrix size = 256 × 256 mm^2^ in the Magnetom Vida scanner.

We resampled the image inputs into a grid of 256 × 256 × 256 voxels with an isotropic spatial resolution of 1 × 1 × 1 mm^3^ using the mri_convert routine in FreeSurfer^23^. Then we extracted 2D coronal slices around the medial temporal lobe through two stages of rigid transformation using DBAD. In the first stage of rigid transformation, DBAD matched the position of the input images to a Korean normal elderly template (KNE) constructed from a cognitively normal elderly population^24^. Using the template-registered input image, DBAD extracted the brain parenchyma using the custom brain extraction algorithm, which is based on a 3D UNet generated by the Brain Extraction Tool in the FMRIB Software Library^25^. In the second stage of rigid transformation, DBAD registered the output images of the first stage to a skull-stripped version of the KNE and extracted 256 2D coronal slices. Based on the selection criteria of the medial temporal lobe atrophy visual rating scale^26^, DBAD selected 30 consecutive 2D coronal slices, starting from the corpus of the hippocampus, that were used as the input images in the DBAD CNN-based model for diagnosing AD. DBAD applied min–max normalization to bound the values of the images between zero and one in all slices^22^.

### MRI-based diagnosis

**Figure 1:**
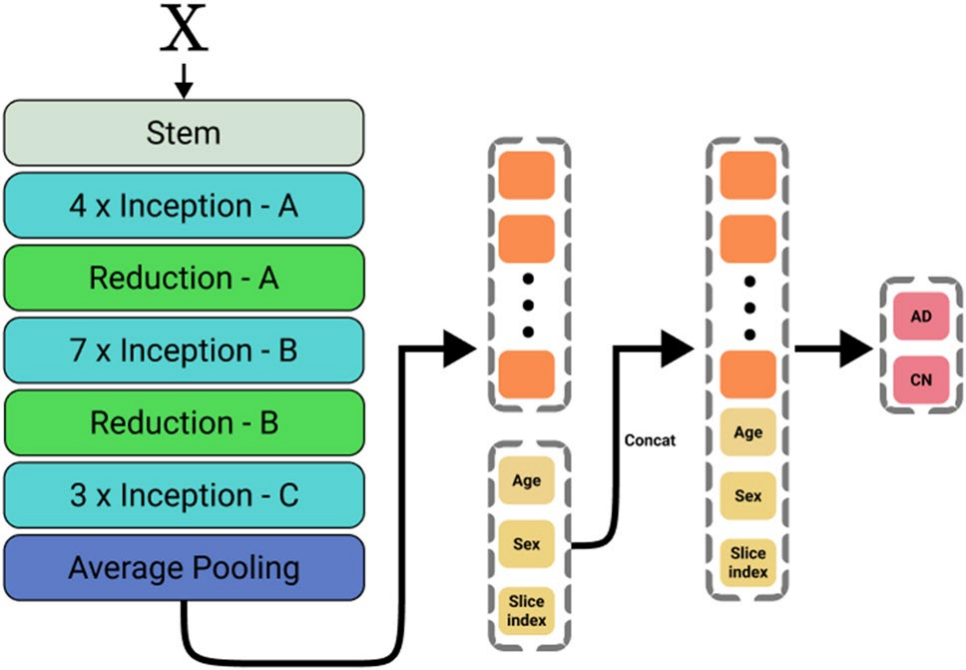
Diagram of the network architecture. For each participant, the model fed 1 out of 30 coronal slices individually. The results of the 30 slices were averaged to produce probability of diagnosing AD for that participants.

The DBAD fed 30 2D coronal slices of medial temporal lobe with age and sex of each participant into the DBAD CNN-based model for diagnosing AD, The DBAD CNN-based model for diagnosing AD uses F Inception-V4 as its backbone and extracts various features that include structural and textural information of the input images. The DBAD CNN-based model for diagnosing AD concatenated age, sex, and the location information of the coronal slices (slice number) and entered them into a fully connected network that calculates the probability of AD of each slice. The DBAD CNN-based model for diagnosing AD averaged the probabilities of AD of the slices to calculate a final score that represents the subject’s probability of having AD (DBAD score, score ranges from 0 to 1). Then we classified the participants with the DBAD score of ≥ 0.38 as AD (DBAD-AD), and those with the DBAD score of < 0.38 as normal (DBAD-CT) based on our previous study^22^ (Fig. 1). Deep learning model was implemented using Pytorch (v.0.4.1) and it was conducted using NVIDIA Geforce GTX 1080 Ti GPU.

Three medical experts (two neuroradiologists and one neurologist) also classified each participant into AD (ME-AD) and normal (ME-CT) groups using full volumetric, T1-weighted MRI scans as well as participant’s age and sex. Each medical expert was blinded to the decisions of other medical experts (ME) and DBAD. We considered the diagnosis that two or more ME agreed upon the consensus diagnosis of the panel.

### Acquisition of ^18^F-florbetaben amyloid brain PET scans and determination of amyloid positivity

Brain ^18^F-florbetaben amyloid PET images were obtained using a Discovery VCT scanner (General Electric Medical Systems; Milwaukee, WI, USA). We injected 8.1 mCi (300 MBq) of ^18^F-florbetaben (Neuraceq, Piramal, Mumbai, India) as a slow single intravenous bolus (6 s/mL) in a total volume of up to 10 mL and obtained 20-min PET images comprising four 5-min dynamic frames after a 90-min uptake period. Trained radiologists with expertise in nuclear medicine determined amyloid beta peptide (Aβ)-positivity based on the brain amyloid plaque load (BAPL) score. The BAPL score is a predefined three-grade scoring system wherein measurements are made by the physician according to the visual assessment of the participant’s amyloid deposition in the brain using Neuraceq. BAPL scores of 1 (BAPL 1), 2 (BAPL 2), and 3 (BAPL 3) indicate no Aβ load, minor Aβ load, and significant Aβ load, respectively. Therefore, BAPL 1 indicates an Aβ-negative status, whereas BAPL 2 and BAPL 3 indicate an Aβ-positive status^27^.

### Standard diagnosis

Geriatric neuropsychiatrists with expertise in dementia research (not participating in the MRI-based diagnoses) administered a face-to-face standardized diagnostic interview as well as physical and neurological examinations using the Korean version of the Consortium to Establish a Registry for Alzheimer’s Disease Assessment Packet Clinical Assessment Battery (CERAD-K-C)^28^ to diagnose cognitive disorders. Laboratory tests, including complete blood counts, chemistry profiles, and serological tests for syphilis, were performed for each participant. Research neuropsychologists, geriatric psychiatrists, and trained research nurses blinded to the MRI-based diagnoses administered the CERAD-K-N, which consists of the following neuropsychological tests: Verbal Fluency Test, 15-item Boston Naming Test, Mini-Mental State Examination (MMSE), Word List Memory Test, Constructional Praxis Test, Word List Recall Test, Word List Recognition Test, Constructional Recall Test, Trail Making Test A/B, Digit Span Test, and Frontal Assessment Battery^28,29^.

We then determined the standard diagnosis and Clinical Dementia Rating (CDR) of each participant at the diagnostic conference in which four research geriatric psychiatrists participated. We diagnosed mild cognitive impairment (MCI) according to the consensus criteria from the International Working Group on MCI^30^; dementia according to the Diagnostic and Statistical Manual of Mental Disorders, Fourth Edition (DSM-IV) diagnostic criteria^31^; and AD according to the National Institute on Aging-Alzheimer’s Association workgroups (NIA-AA) criteria^32^. All AD patients were Aβ-positive on ^18^F-florbetaben brain PET scan and met the NIA-AA criteria of preclinical AD^33^ and MCI or dementia due to AD^34,35^.

### Statistical analysis

We compared the continuous and categorical variables between groups using Mann–Whitney U-test and Pearson chi-square test, respectively. We then compared the diagnostic classification between DBAD and panel of medical experts (ME). We estimated sensitivity, specificity and accuracy of the diagnoses made by the DBAD and the ME using the receiver operating characteristic (ROC) curve analysis. Then, we compared the classification performance of the DBAD and the ME using McNemar’s test.

All analyses were performed using SPSS for Windows (version 25.0; IBM Co., Armonk, NY, USA) and MedCalc version 16.4.3 (MedCalc Software, Mariakerke, Belgium). We considered two-sided *p*-values < 0.05 to indicate statistical significance.

## Results

The demographic characteristics of the participants are summarized in Table 1. The ME-AD and DBAD-AD groups were older (*p* < 0.001) and less educated than the ME-CT (*p* = 0.016) and DBAD-CT (*p* < 0.001) groups, respectively (Table 1).

**Table 1:** Demographic characteristics of the participants.

	All	DBAD			Panel of medical experts		
		AD (N = 37)	Normal (N = 61)	*p*^†^	AD (N = 36)	Normal (N = 62)	*p*^†^
Age (years)	70.6 ± 6.9	74.7 ± 6.2	68.1 ± 6.1	< 0.001	74.9 ± 6.2	68.1 ± 6.1	< 0.001
Women (%)	62.2	70.3	57.4	0.202	50.0	69.4	0.057
Education (years)	12.5 ± 4.9	10.3 ± 4.3	13.8 ± 4.8	< 0.001	10.9 ± 4.3	13.4 ± 5.0	0.016
**CDR, n (%)**							
0	44 (44.9)	4 (10.8)	40 (65.6)	< 0.001	6 (16.7)	38 (61.3)	< 0.001
0.5	38 (38.8)	20 (54.1)	19 (31.1)		17 (47.2)	22 (35.5)	
1	7 (7.1)	5 (13.5)	2 (3.3)		5 (13.9)	2 (3.2)	
≥ 2	9 (9.2)	8 (21.6)	0 (0.0)		8 (22.2)	0 (0.0)	

Continuous variables are presented as mean ± standard deviation.

*DBAD* VUNO Med-DeepBrain AD, *AD* Alzheimer’s disease, *CDR* Clinical Dementia Rating.

^†^Student’s t-test was used for continuous variables and the chi-square test for categorical variables.

As summarized in Table 2, the ME classified 36 participants as ME-AD and the rest as ME-CT. The decisions of the three medical experts completely matched for 77 participants (26 ME-AD and 51 ME-CT), and their κ agreement was 0.694 (*p* < 0.001). Among the 36 ME-AD participants, 15 were diagnosed with AD in the standard diagnostic assessment (12 dementia due to AD, one MCI due to AD, and two preclinical AD). Among the 21 ME-AD participants who were not diagnosed with AD in the standard diagnostic assessment, 6 were diagnosed with other types of dementia (4 with subcortical vascular dementia and 2 with suspected non-AD pathophysiology [SNAP]), 7 with Aβ-negative MCI, and 8 were Aβ-negative and cognitively normal. Among the 62 ME-CT participants, 47 were Aβ-negative and cognitively normal according to the standard diagnostic assessment. In contrast, among the remaining 15 participants, 9 were diagnosed with AD (three with dementia due to AD, three with MCI due to AD, and three with preclinical AD), 1 with SNAP, and 5 with Aβ-negative MCI.

**Table 2:** Results of standardized clinical assessment of Deep Brain AD and medical experts.

	DBAD		Panel of medical experts	
	AD (N = 37)	Normal (N = 61)	AD (N = 36)	Normal (N = 62)
**Standard diagnosis**				
Aβ-positive				
Preclinical AD	1 (2.7)	4 (6.6)	2 (5.6)	3 (4.8)
MCI due to AD	2 (5.4)	2 (3.3)	1 (2.8)	3 (4.8)
Dementia due to AD	14 (37.8)	1 (1.6)	12 (33.3)	3 (4.8)
All	17 (45.9)	7 (11.5)	15 (41.7)	9 (14.5)
Aβ-negative				
Normal	8 (21.6)	47 (77.0)	8 (22.2)	47 (75.8)
MCI due to non-AD	6 (16.2)	6 (9.8)	7 (19.4)	5 (8.1)
Dementia due to non-AD	6 (16.2)	1 (1.6)	6 (16.7)	1 (1.6)
All	20 (54.1)	54 (88.5)	21 (58.3)	53 (85.5)

*DBAD* VUNO Med-DeepBrain AD, *AD* Alzheimer’s disease, *MCI* mild cognitive impairment.

Number of cases with percentage in parenthesis.

The DBAD classified 37 participants as DBAD-AD and the rest as DBAD-CT. Among the 37 DBAD-AD participants, 17 were finally diagnosed with AD in the standard diagnostic assessment (14 with dementia due to AD, 2 with MCI due to AD, and 1 with preclinical AD). Among the 20 DBAD-AD participants who were not diagnosed with AD in the standard diagnostic assessment, 6 were diagnosed with other types of dementia (4 subcortical vascular dementia, 2 SNAP), 6 with Aβ-negative MCI, and 8 were Aβ-negative and cognitively normal. Among the 61 DBAD-CT participants, 47 were Aβ-negative or cognitively normal according to the standard diagnostic assessment, whereas in the remaining 14 DBAD-CT participants, 7 were diagnosed with AD (1 dementia due to AD, 2 MCI due to AD, and 4 preclinical AD), 1 with SNAP, and 6 with Aβ-negative MCI (Table 2).

As summarized in Table 3, the accuracy for classifying Aβ-positive AD patients from Aβ-negative cognitively normal controls was comparable between DBAD and the ME consensus diagnosis. The specificity of classifying Aβ-negative cognitively normal controls as normal was the same for DBAD and ME. The sensitivity of DBAD in diagnosing Aβ-positive AD patients was higher than that of ME at all levels of the definition of AD, although the differences were not significant. However, when each ME diagnosis was analyzed separately, the accuracy of DBAD was higher than that of one ME (*p* = 0.031 for dementia due to AD, *p* = 0.009 for dementia/MCI due to AD, and *p* = 0.016 for dementia/MCI/preclinical due to AD).

**Table 3:** Diagnostic performance for classifying amyloid β-positive Alzheimer’s disease from amyloid β-negative cognitively normal controls.

	DBAD	Diagnosis of the medical expert (ME)				Statistics^†^			
		ME 1 ^a^	ME 2 ^b^	ME 3 ^c^	Consensus ^d^	a	b	c	d
**Accuracy**									
Dementia due to AD	87.1 (77.0, 93.9)	84.3 (73.6, 91.9)	84.3 (73.6, 919)	78.6 (67.1, 87.5)	84.3 (73.6, 91.9)	0.500	0.500	0.031	0.500
Dementia/MCI due to AD	85.1 (75.0, 92.3)	81.1 (70.3, 89.3)	81.1 (70.3, 89.3)	74.3 (62.8, 83.8)	81.1 (70.3, 89.3)	0.250	0.250	0.009	0.250
Dementia/MCI/preclinical due to AD	81.0 (70.6, 89.0)	78.5 (67.8, 86.9)	77.2 (66.4, 85.9)	72.2 (60.9, 81.7)	78.5 (67.8, 86.9)	0.500	0.250	0.016	0.500
**Sensitivity**									
Dementia due to AD	93.3 (68.1, 99.8)	80.0 (51.9, 95.7)	73.3 (44.9, 92.2)	86.7 (59.5, 98.3)	80.0 (51.9, 95.7)	0.500	0.250	1.000	0.500
Dementia/MCI due to AD	84.2 (60.4, 96.6)	68.4 (43.5, 87.4)	63.2 (38.4, 83.7)	68.4 (43.5, 87.4)	68.4 (43.5, 87.4)	0.376	0.219	0.250	0.376
Dementia/MCI/preclinical due to AD	70.8 (48.9, 87.4)	62.5 (40.6, 81.2)	54.2 (32.8, 74.4)	62.5 (40.6, 81.2)	62.5 (40.6, 81.2)	0.688	0.219	0.625	0.688
**Specificity**									
Dementia due to AD	85.5 (73.3, 93.5)	85.5 (73.3, 93.5)	87.3 (75.5, 94.7)	76.4 (63.0, 86.8)	85.5 (73.3, 93.5)	1.000	1.000	0.267	1.000
Dementia/MCI due to AD	85.5 (73.3, 93.5)	85.5 (73.3, 93.5)	87.3 (75.5, 94.7)	76.4 (63.0, 86.8)	85.5 (73.3, 93.5)	1.000	1.000	0.267	1.000
Dementia/MCI/preclinical due to AD	85.5 (73.3, 93.5)	85.5 (73.3, 93.5)	87.3 (75.5, 94.7)	76.4 (63.0, 86.8)	85.5 (73.3, 93.5)	1.000	1.000	0.267	1.000

*DBAD* VUNO Med-DeepBrain AD, *ME* Medical Expert, *AD* Alzheimer’s disease, *MCI* mild cognitive impairment.

^†^McNemar’s test compared with the diagnosis of DBAD.

As summarized in Table 4, the accuracy of distinguishing Aβ-positive AD patients from Aβ-negative non-AD patients and the specificity for classifying Aβ-negative participants as non-AD were also comparable between the DBAD and ME groups. The sensitivity of DBAD in diagnosing Aβ-positive AD patients was higher than that of ME at all levels of the definition of AD, but the differences were not significant (Table 4). The DBAD accuracy of distinguishing Aβ-positive AD patients from Aβ-negative non-AD participants was lower than that of distinguishing Aβ-positive AD patients from Aβ-negative cognitively normal controls, because the specificity for classifying Aβ-negative participants as non-AD was lower than that for classifying Aβ-negative cognitively normal controls as normal.

**Table 4:** Diagnostic performance for classifying amyloid β-positive Alzheimer’s disease from amyloid β-negative controls.

	DBAD	Medical experts	*p*^†^
**Accuracy**			
Dementia due to AD	76.4 (66.2, 84.8)	73.0 (62.6, 81.9)	0.581
Dementia/MCI due to AD	75.3 (65.2, 83.6)	71.0 (60.6, 79.9)	0.455
Dementia/MCI/preclinical due to AD	72.4 (62.5, 81.0)	69.4 (59.3, 78.3)	0.629
**Sensitivity**			
Dementia due to AD	93.3 (68.1, 99.8)	80.0 (51.9, 95.7)	0.500
Dementia/MCI due to AD	84.2 (60.4, 96.6)	68.4 (43.5, 87.4)	0.375
Dementia/MCI/preclinical due to AD	70.8 (48.9, 87.4)	62.5 (40.6, 81.2)	0.688
**Specificity**			
Dementia due to AD	73.0 (61.4, 82.6)	71.6 (59.9, 81.5)	1.000
Dementia/MCI due to AD	73.0 (61.4, 82.6)	71.6 (59.9, 81.5)	1.000
Dementia/MCI/preclinical due to AD	73.0 (61.4, 82.6)	71.6 (59.9, 81.5)	1.000

*DBAD* VUNO Med-DeepBrain AD, *AD* Alzheimer’s disease, *MCI* mild cognitive impairment.

^†^McNemar’s test compared with the diagnosis of DBAD.

## Discussion

This study demonstrated that DBAD can support the decision on the diagnosis of AD in the hospitals where medical specialists on AD are not available by showing that the diagnostic accuracy for AD of DBAD was comparable to that of the medical experts on AD who are working at the referral hospitals.

Medial temporal lobe atrophy (MTA) is now considered a valid diagnostic marker of AD at the MCI stage^36^, and the MTA rating is widely used in clinical practice to determine the presence of AD-related neurodegeneration^22^. The DBAD uses 30 coronal slices starting from the corpus of the hippocampus based on the Scheltens score for MTA ratings^22,26^, whereas an ME panel can use additional information such as cortical atrophy patterns, ventricular enlargement, or small vessel disease through the full volume of T1-weighted MRI. Despite this disparity in the information provided, DBAD demonstrated an expert level of diagnostic accuracy and was superior to individual ME diagnosis.

The diagnostic accuracy and sensitivity of both DBAD and the ME were better for dementia due to AD than for MCI due to AD and/or preclinical AD. DBAD is an algorithm developed and validated using a divided dataset consisting of normal controls and dementia due to mild AD (CDR 0.5 or 1) for typical normal and AD classification^22^. Therefore, the diagnostic performance of DBAD may be lower when diagnosing preclinical AD or MCI due to AD than when diagnosing dementia due to AD. MTA is less pronounced in MCI and preclinical AD than in dementia due to AD^37^. The University of California-Los Angeles Alzheimer’s Disease Research Center has studied the hippocampal volume loss between MCI due to AD and dementia due to AD using 20 follow-up MCI participants and revealed that the annual atrophy rate for those who remained in the MCI stage due to AD was 2.8%, while that for those who developed dementia due to AD was 3.7%^38^. Moreover, the Mayo Clinic AD Research Center/AD Patient Registry also studied the hippocampal volume loss of normal controls, patients with MCI, and those with probable AD in a sample of 129 participants and found that the mean annualized rates of hippocampal volume loss were 1.73% for normal controls, 2.5% for MCI, and 3.5% for probable AD^39^. This body of literature implies that MTA is a later event in AD progression^40^, which may also explain the lower diagnostic performance of DBAD when including preclinical and MCI stages of AD than when only including dementia due to AD. DBAD exploits image slices that represent MTA in the coronal view from 3D T1-weighted MRI to diagnose AD. However, DL using MRI not only helps interpret and diagnose the volume reduction or atrophy of the brain but also provides a comprehensive reflection of other sensitive features (e.g., MRI texture^41^) related to AD that DBAD may have analyzed during diagnosis.

Nevertheless, even after including preclinical AD and MCI due to AD, the diagnostic performance of DBAD was comparable to that of ME. This supports the promising role of DBAD as a diagnostic assistant tool for AD, even in the early stages of AD in a clinical setting in the absence of an ME. In addition, the diagnostic accuracy, sensitivity, and specificity of DBAD in diagnosing AD showed a similar level of performance to that in the previous paper for algorithm development^22^. This study may also serve as an independent validation study for the diagnostic accuracy, sensitivity, and specificity of DBAD.

Various studies on DL algorithms to diagnose AD have been conducted^12,13,16–18^; however, few algorithms have been validated by comparisons of DL algorithm performance with that of medical doctors. Qiu et al.^42^ created an algorithm for diagnosing AD using DL with the 417 Alzheimer’s Disease Neuroimaging Initiative dataset. They compared the diagnostic performance of the developed algorithm and that of neurologists after they were provided with full volumetric T1-weighted MRI scans, age, sex, and MMSE scores of the participants. In line with our study, their DL model (accuracy, 0.834 ± 0.020) outperformed the neurologists (accuracy, 0.823 ± 0.094). In addition, a study by Nagendran^43^ gathered the research that compared the diagnostic performance of DL algorithms and clinicians, based on medical imaging for diseases such as cancer, cataract, and colon polyps. Among a total of 77 papers comparing DL and clinicians’ diagnoses, 30% studies showed that DL performance was superior to clinicians’, 17% said DL was comparable or better, 32% said DL was comparable, 18% said DL was able to help a clinician perform better, and 3% said DL was not superior. These previous studies suggest the usefulness of the DL algorithm as a supporting tool in clinical settings.

The current study has some limitations. First, the number of ME included was limited, and a larger number of ME are needed for further validation studies. Second, although DBAD showed comparable performance to that of ME, we would consider incorporating other AD signature regions, such as the precuneus^44,45^ and posterior cingulate cortex^46,47^ to the DBAD algorithm to enhance the diagnostic accuracy even in the preclinical AD stage. However, in this case, we may have to sacrifice the current strength of DBAD processing speed.

In this study, we validated the diagnostic performance of AD classification CNN-based algorithm by comparing it with MRI diagnosis by medical experts. The VUNO Med-DeepBrain AD may support the clinical decision of the physicians who are not specialized in AD, which may enhance the accessibility of AD diagnosis and treatment.

## Data Availability

Data are not publicly available because they contain sensitive participant information. Individual, de-identified, participant data that underlie the results reported in this article may be made available to qualified researchers upon reasonable request. Proposals should be directed to K.W.K. to gain access.
